# Visuospatial working memory capacity moderates the relationship between anxiety and OCD related checking behaviors

**DOI:** 10.3389/fpsyt.2022.1039849

**Published:** 2023-01-09

**Authors:** Pengchong Wang, Zijun Yan, Tao Chen, Wenwen Cao, Xiangyun Yang, Fanqiang Meng, Yuqing Liu, Zhanjiang Li

**Affiliations:** ^1^Beijing Key Laboratory of Mental Disorders, National Clinical Research Center for Mental Disorders, Beijing Anding Hospital, Capital Medical University, Beijing, China; ^2^Advanced Innovation Center for Human Brain Protection, Capital Medical University, Beijing, China; ^3^Brain and Mind Centre, The University of Sydney, Sydney, NSW, Australia; ^4^School of Psychology, The University of Sydney, Sydney, NSW, Australia

**Keywords:** obsessive-compulsive disorder, checking behavior, cognitive flexibility, anxiety, moderation

## Abstract

**Background:**

Compulsive checking behavior is the most prevalent compulsive behavior in patients with obsessive-compulsive disorder (OCD). While some studies have shown that anxiety and executive function influence compulsive checking behavior, the relationship between these constructs is inconclusive. Hence, we sought to explore the interplay between executive function, anxiety and compulsive checking behavior.

**Materials and methods:**

47 healthy participants (HC) and 51 patients with OCD participated in the study. Symptoms and emotional states were assessed using the Yale-Brown Obsessive Compulsive Scale, the Obsessive-Compulsive Inventory-Revised, the Beck Anxiety Inventory, and the Beck Depression Inventory. Participants also completed three tests of neuropsychological functioning: the Stop Signal Task, the Spatial working memory Task, and the Wisconsin card sorting test. We analyzed the relationships between anxiety, executive function, and compulsive checking symptoms.

**Results:**

Patients with OCD showed significantly greater anxiety (*p* < 0.001) and impairments in visuospatial working memory function (*p* = 0.030) compared to HC participants, while inhibition and set-shifting were not significantly different between the two groups. Visuospatial working memory was negatively related to compulsive checking behavior (*p* = 0.016). Visuospatial working memory also played a moderating role in the positive relationship between anxiety and compulsive checking behavior (β = −0.281, *p* = 0.022).

**Conclusion:**

Anxiety symptoms play an important role in explaining compulsive checking behavior in patients with OCD who have relatively weak visuospatial working memory ability. These findings provide a foundation for further research regarding the roles of emotion and cognitive inflexibility in compulsive checking behavior in patients with OCD.

## 1. Introduction

Obsessive-compulsive disorder (OCD) is a condition characterized by persistent, intrusive obsessions, and repetitive compulsions. Symptoms of OCD are heterogeneous (1) and various subtypes of the condition have been identified (2), such as checking, washing, ordering, and hoarding. Compulsive checking behavior is the most prevalent compulsive behavior in individuals with OCD (3). Numerous studies have shown that repetitive checking behavior breeds doubt (4), uncertainty (5), reduces memory confidence (6, 7), and impairs inhibition (8). Although the consequences of compulsive checking behavior have been widely studied, the factors inducing or maintaining checking behavior remain unclear (9).

According to the dimensional account of OCD (10), subtypes of OCD arise from the interaction of three main factors: emotional vulnerability (typically anxiety and depression), cognitive inflexibility, and an imbalance in goal-directed behavior and habitual control. This interaction is assumed to be at the core of all OCD subtypes. However, the interaction between these factors and the role they play in compulsive checking behavior is unclear, and requires consideration. Based on the cognitive theory of compulsive checking (11) and the cognitive flexibility hypothesis (12), anxiety and cognitive inflexibility are the typical emotional and cognition vulnerability factors of OCD and are closely related to compulsive checking behavior. Therefore, a better understanding of these factors’ interaction underlying compulsive checking subtype may provide insights into the mechanisms underpinning OCD.

### 1.1. Anxiety and compulsive checking behavior

Anxiety symptoms are the central element of OCD and may affect the development and maintenance of obsessive-compulsive symptoms in several ways. For instance, anxiety disorders are one of the most frequently found comorbid psychiatric disorders in OCD (13, 14), and anxiety is significantly associated with more severe obsessive-compulsive symptoms and other key OCD symptoms (i.e., negative appraisals of intrusive thoughts) (15).

Furthermore, anxiety symptoms may also contribute to reassurance-seeking behaviors, such as compulsive checking. According to cognitive theory of checking (11), the perception of possible harm results in increased anxiety or discomfort, which leads patients with OCD to engage in compulsive checking to obtain relief from their distress and/or anxiety. For instance, Wake et al. (16) reported that greater self-reported anxiety was associated with higher subjective ratings of check-up impulsivity during the Visual Discrimination and Checking Task. Similarly, anxiety symptoms have also been shown to significantly affect checking behavior (17), while evidence from network analysis suggests that doubting/checking symptoms are linked to generalized anxiety symptoms (i.e., worry, rumination).

### 1.2. Cognitive flexibility and compulsive checking behavior

Cognitive flexibility is a mental ability to adjust to change by switching or shifting from thinking about one conceptual representation to another (18). The researchers found that impaired cognitive flexibility can worsen symptoms by affecting the regulation and control of the mind (10).

Both clinical observation and neurocognitive studies demonstrate behavioral and neurobiological deficits in cognitive flexibility in OCD patients, and the impairment of cognitive flexibility in OCD may result from deficits in a range of executive function components, such as inhibition (12), working memory and set-shifting (19).

Recent studies have suggested compulsive checking symptoms are associated with impairments in executive function subcomponents. It has been reported that compulsive checking was significantly associated with poorer inhibition on the Trail Making test (20). Several studies have also reported that patients with OCD have impaired visuospatial working memory (VWM) (5, 21), especially in those with compulsive checking symptoms (4). Based on the outcome of a meta-analysis, Leopold and Backenstrass (22) reported that checkers were significantly more impaired in set-shifting than washers. Conversely, some studies have not found impaired set-shifting in patients with checking behavior (23, 24).

These findings underscore that anxiety symptom and cognitive inflexibility may play as important vulnerability factors in checking subtype of OCD, however, their individual and shared impact on compulsive checking symptoms have rarely been studied.

### 1.3. The interplay between anxiety and cognitive flexibility

Traditionally, compulsive checking behavior is viewed as an anxiety-driven behavior that could neutralize, prevent, or reduce anxiety immediately. More recently, however, Hirsch and Mathews (25) indicated that pathological anxiety is largely sustained by impairments in cognitive control, particularly within the context of negative emotional information (26). Similarly, Pruessner et al. (27) have proposed the cognitive control framework of emotion regulation flexibility, which suggests that emotion regulation is associated with inhibition, updating, and shifting functions.

Evidence has also been reported to show that anxiety can interfere with cognitive functioning (28) and affect goal-directed or impulsive behaviors. Working memory plays a key role in the cognitive problems experienced by anxious people by limiting the resources needed to perform goal-directed tasks (28–30). Yu et al. (31) reported that cognitive flexibility played a mediating role between anxiety and impulsivity, and moderated the effects of anxiety on motor impulsivity. Given that compulsive checking behavior is associated with both anxiety and cognitive flexibility, it is conceivable that there might be an interplay between anxiety and cognitive flexibility in the pathophysiology of compulsive checking behavior.

Previous studies have shown that anxiety can predict the severity of compulsive checking behaviors, and the severity of the symptoms is related to executive function. It also suggests that executive function may moderate the relationship between anxiety and compulsive checking behaviors. But a definitive conclusion is still lacking. To further our understanding of these relationships, we assessed the three core components of executive function and anxiety symptoms in the present study. We sought to explore cognitive flexibility in patients with OCD and assess which components might interact with anxiety to affect the severity of compulsive checking behavior.

## 2. Materials and methods

### 2.1. Participants

Considering the impairment of executive function in adolescents and elder is different from that in adults, to prevent the influence of age, only adult healthy participants (HC) and patients with OCD were invited to participate in present study. Patients were recruited from the outpatient department of Beijing Anding Hospital. The inclusion criteria for patients with OCD were: (1) Age 18–45 years, and a junior high school education or above; (2) meeting the diagnostic criteria for obsessive-compulsive disorder as specified in the Diagnostic and Statistical Manual of Mental Disorders, fourth edition (DSM-IV); (3) With at least mild level of symptom severity, the Yale-Brown obsessive compulsive scale (Y-BOCS) total score ≥ 8; (4) right-handedness, normal or corrected vision, no color blindness or weakness. The exclusion criteria were: (1) meeting the DSM-IV diagnostic criteria for any comorbid mental disorder such as schizophrenia or mood disorder, (2) having received convulsion-free electroconvulsive therapy, neuromodulation, and other physical therapy within the last 4 weeks; (3) a history of brain organic disease and/or major somatic disease; and (4) evidence of drug dependence and use of psychoactive substances.

Healthy participants were recruited *via* advertising. The inclusion criteria for HC were (1) age 18–45 years old, junior high school education or above; (2) Without clinically significant anxiety or depression symptom, a Beck Anxiety Inventory (BAI) score < 15 points, a Beck Depression Inventory (BDI) score < 15 points; (3) right handedness, normal or corrected vision, no color blindness or weakness. The exclusion criteria were: (1) a DSM-IV diagnosis of obsessive-compulsive disorder, schizophrenia, mood disorder, and other mental disorders or a previous diagnosis of OCD; (2) a history of brain organic disease and/or major somatic disease; and (3) evidence of drug dependence and use of psychoactive substances.

### 2.2. Procedure

In the current experiment, three executive function tasks were designed using E-Prime 2.0 software (Psychology Software Tools Ltd., Pittsburgh, PA, USA). Visual stimuli were presented using a screen resolution of 800 × 600 with a 60 Hertz refresh rate. Participants sat approximately 60 cm from the computer screen. After completing the clinical assessments, participants were asked to complete the executive function tasks on the computer. In addition to the three executive function tasks, participants also performed sustained attention to response tasks and other interventions. The study was completed in March 2021–April 2022.

### 2.3. Measurements

The Mini-International Neuropsychiatric Interview (MINI) was used to screen out other mental diseases by one trained researcher. The severity of obsessive-compulsive symptoms was evaluated by the Yale-Brown Obsessive-Compulsive Scale (Y-BOCS), which includes two aspects of OCD: obsessions (Items 1–5) and compulsions (Items 6–10) (32). The Chinese version of the Y-BOCS has good interrater reliability (*r* = 0.75) and test-retest reliability (*r* = 0.91), as well as good construct validity (33). Obsessive-Compulsive Inventory-Revised (OCI-R) (34) was used to measure self-reported obsessive compulsive symptom, which includes 18 items, such as washing, obsessing, hoarding, ordering, checking, and neutralizing, mixed with six dimensions to assess obsessive-compulsive symptoms. The Chinese version of OCI-R shows good internal consistency (Cronbach’s α = 0.84) and test–retest reliability (*r* = 0.96) (35). BAI (36) and the BDI (37) were applied to measure self-evaluated anxiety and depression level. The Chinese version of the BAI has demonstrated excellent internal consistency (Cronbach’s α = 0.95) (38), and the Chinese version of the BDI-II has demonstrated good internal consistency (Cronbach’s α = 0.94) and test-retest coefficients (*r* = 0.55) (37).

### 2.4. Executive function assessment

#### 2.4.1. Stop signal task

The SST includes response and stop tasks to measure cognitive flexibility (39). A fixation point “+” initially appeared on the center of the screen, which was quickly followed by (after 500 ms), a square or circle (response signal) which appeared for 1,000 ms. Participants were asked to make a quick and selective response, and abort the answer previously displayed when a subsequently presented red star (stop signal) was displayed. The time between the presentation of the imperative stimulus and the presentation of the stop signal is termed the “stop signal delay” (SSD); SSD values range from 50 to 950 ms, with the initial SSD time being 250 ms. The delay time was adjusted according to participants’ responses, with correct responses increasing the delay by 50 ms, and incorrect responses decreasing the delay by 50 ms. SST consist a total 160 trials: the ratio of go trial to stop trial is 3:1. SSD after reaction (minimum 50, maximum 950) vary according to the correct or wrong stop times of the subject, correct + 50, error − 50, initial value 250. The stop signal reaction time (SST_SSRT_) is the index of inhibitory control.

#### 2.4.2. Spatial working memory task

Participants were also asked to perform a computerized spatial working memory task (40). A 5 × 5 gray square was initially displayed on the screen, followed by the presentation of red squares that appeared randomly at each of 25 positions. Participants were asked to remember and click in the sequence where the red squares had appeared. The number of the target red square increased in turn from 2. After three consecutive selections, the span of the target square increased by 1, with a maximum of 6. The number of red squares selected correctly (i.e., the capacity of VWM) was recorded. The working memory index in this task is visuospatial memory capacity (VWM_*capacity*_).

#### 2.4.3. Wisconsin card sorting test

The WCST was used to measure cognitive flexibility, and includes four stimulus cards and 128 response cards, each painted with 1–4 triangles, stars, crosses, or circles in red, green, blue, and yellow, respectively (41). Among them, four stimulus cards are pictures with one red triangle, two green stars, three yellow crosses, and four blue circles. According to the rules, participants are asked to accurately sort every response card according to one of four stimulus cards by providing feedback regarding their response (correct or incorrect). The sorting rule changes after ten correct matches, which occurs without warning to the participant. The test will automatically end when the subject has completed three groups (color, shape, quantity) of classification, or has used up all 128 cards. The whole test is 128 times, about 10 min. The WCST’s index of set-shifting is the rate of perseverative errors (WCST_*Rpe*_).

### 2.5. Data analysis

IBM SPSS Statistics 26.0 software and Mplus8.3 were used for statistical analysis. The demographic and clinical data of the two groups were compared by independent sample *t*-tests and chi-square tests. The Pearson correlation coefficient was used to analyze the relationship between each of the three task indices and the severity of compulsive checking symptoms. Mplus8.3 was used to analyze whether the various executive functioning components (SST_SSRT_, VWM_capacity_, WCST_Rpe_) moderated the relationship between anxiety symptoms and compulsive checking symptoms. Two-tailed tests were performed in all analyses, and the significance level was 0.05. Pauta criterion was applied to outlier detection of reaction time on task, and it is assumed that data exceeding three standard deviation of the sample mean is outlier. Cohen’s *d* was calculated to reflect the effect sizes of statistical result (42).

## 3. Results

### 3.1. Demographic and clinical data analysis

A total of 47 HC and 51 patients with OCD were enrolled in the study. One OCD and one HC participant was excluded as they could not complete the Wisconsin card sorting test; one OCD and two HC participants were excluded as reaction time scores were below or above three SD of the group’s mean, resulting in a final sample size of *n* = 49 for OCD group and *n* = 44 for HC group.

The average age of OCD was 29.33 and of HC was 28.89 years, and the number of male and female participants in each group was approximately equal. There were no significant differences in gender, age, and education level between HC and patients with OCD (*p* > 0.05). Of the 49 OCD participants, 20 (40.8%) had never been prescribed medications or had stopped taking medications for at least 4 weeks before participating in this study, 18 (36.7%) have been receiving the SSRI therapy and 11 (22.4%) have been receiving multi type of prescribed medications.

The mean score of Y-BOCS in patients with OCD was 19.73 ± 7.25, and the scores for depression and anxiety were significantly higher than those of HC (*p* < 0.001) (Table 1).

**TABLE 1:** Demographic characteristics and clinical symptoms of the two groups.

Variables	HC	OCD	*t*/χ^2^	*p*	Cohen’s *d*
	(*n* = 44)	(*n* = 49)			
Gender			0.164^a^	0.686	0.084
Male	22 (50%)	26 (53%)			
Female	22 (50%)	23 (47%)			
Age, M (SD)	28.89 (8.57)	29.33 (6.39)	−0.283	0.778	0.059
Education			6.487^a^	0.090	0.548
Junior high school	0	2			
Senior high school	5	5			
Undergraduate	31	24			
Master’s degree or above	8	18			
BAI	2.18 (2.90)	13.63 (10.48)	−7.010	< 0.001	1.455
BDI	3.66 (4.52)	13.78 (9.87)	−6.236	< 0.001	1.295
OCI-R	2.34 (3.35)	22.02 (14.32)	−8.894	< 0.001	1.848
OCI-R-checking	0.34 (1.01)	3.57 (3.29)	−6.260	< 0.001	1.298

HC, healthy participants; OCD, obsessive-compulsive disorder participants; BAI, the Beck Anxiety Inventory; BDI, the Beck Depression Inventory; OCI-R-checking, the checking item of Obsessive-Compulsive Inventory-Revised.

### 3.2. Comparison of executive function between the two groups

There were no significant differences in SST_SSRT_, and WCST_Rpe_ between the two groups (*p* > 0.05), although the capacity of VWM was significantly lower in the group with OCD than in the HC group (*p* < 0.05) (Table 2).

**TABLE 2:** Executive function indicators of the two groups.

Variables	HC (*n* = 44)	OCD (*n* = 49)	*t*	*p*	Cohen’s *d*
SST_SSRT_ (ms)	207.11 (84.34)	221.23 (65.10)	-0.908	0.366	0.189
VWM_capacity_ (n)	46.02 (17.31)	38.63 (14.96)	2.208	0.030	–0.459
WCST_Rpe_ (%)	15.95 (14.07)	19.18 (19.45)	-0.908	0.366	0.189

HC, healthy participants; OCD, obsessive-compulsive disorder participants; SST_SSRT_, the stop signal reaction time; VWM_capacity_, visuospatial memory capacity; WCST_Rpe_, the rate of perseverative errors.

### 3.3. Correlation analysis between anxiety, executive function indicators, and checking symptoms

The BAI scores were positively correlated with the severity of general compulsion symptoms measured by Y-BOCS (*p* = 0.007) and checking symptoms as measured by the OCI-R (*p* = 0.011). The three components of executive function were not related to the severity of general compulsion symptoms (*p* > 0.05). VWM capacity was found to negatively correlate with the severity of checking symptoms (*p* = 0.016), while the SST_SSRT_ and WCST_Rpe_ were not related to the severity of checking symptoms (*p* > 0.05) (Table 3).

**TABLE 3:** Correlations between anxiety, executive function indicators, and checking symptoms.

	YBOCS-C	Checking	BAI	SST_SSRT_	VWM_capacity_
BAI	0.380** (0.007)	0.361* (0.011)	/	/	/
SST_SSRT_	−0.261 (0.070)	−0.041 (0.778)	−0.152 (0.298)	/	/
VWM_capacity_	−0.083 (0.572)	−0.343* (0.016)	−0.193 (0.183)	−0.122 (0.405)	/
WCST_Rpe_	0.126 (0.388)	0.041 (0.778)	−0.059 (0.688)	0.162 (0.265)	−0.393** (0.005)

Checking, the checking item of Obsessive-Compulsive Inventory-Revised (OCI-R); BAI, the Beck Anxiety Inventory; SST_SSRT_, the stop signal reaction time; VWM_capacity_, visuospatial memory capacity; WCST_Rpe_, the rate of perseverative errors; YBOCS-C, the compulsion item score of the Yale-Brown Obsessive-Compulsive Scale (Y-BOCS). **P < 0.01 and *P < 0.05.

### 3.4. Moderate effects of executive function on the relationship between anxiety and general compulsion symptom

Hierarchical regression was utilized to explore which components of executive function have moderation effect on the relationship between anxiety and general compulsion symptom.

Data were first standardized. Then, gender, age, and education level were included as covariables. We used levels of anxiety (BAI) as independent variables, the severity of general compulsion symptoms as dependent variables, and three indicators of executive function as moderators. In Model 1, SST_SSRT_ was used as the moderator. VWM_capacity_ was used as the moderator in Model 2, and WCST_Rpe_ was used as the moderator in Model 3.

The results showed that the main effect of anxiety on general compulsion symptoms measured by the Y-BOCS was significant (*p* < 0.05), but the main effects of three components, and their interaction with anxiety level were not significant (*p* > 0.05). It suggested that there were no moderating effects of three components of executive function between anxiety level and general compulsion symptoms (*p* > 0.05) (Table 5).

**TABLE 4:** The moderating effect of executive function on anxiety and general compulsion symptoms.

	Variables	Standardized *β*	S.E.	*p*	Cohen’s *d*
Model 1	BAI	0.353	0.119	0.003	0.881
	SST_SSRT_	−0.215	0.121	0.076	-0.440
	BAI × SST_SSRT_	−0.031	0.122	0.802	-0.062
Model 2	BAI	0.398	0.110	< 0.001	1.002
	VWM_capacity_	0.144	0.117	0.218	0.396
	BAI × VWM_capacity_	−0.196	0.114	0.085	-0.400
Model 3	BAI	0.368	0.126	0.003	0.920
	WCST_Rpe_	−0.030	0.147	0.840	-0.060
	BAI × WCST_Rpe_	0.006	0.131	0.963	0.112

BAI, the Beck Anxiety Inventory; SST_SSRT_, the stop signal reaction time; VWM_capacity_, visuospatial memory capacity; WCST_Rpe_, the rate of perseverative errors.

**TABLE 5:** The moderating effect of executive function on anxiety and checking symptoms.

	Variables	Standardized *β*	S.E.	*p*	Cohen’s *d*
Model 1	BAI	0.350	0.127	0.006	0.873
	SST_SSRT_	−0.017	0.134	0.898	-0.034
	BAI × SST_SSRT_	−0.046	0.134	0.730	-0.092
Model 2	BAI	0.344	0.123	0.005	0.857
	VWM_capacity_	−0.220	0.133	0.099	-0.451
	BAI × VWM_capacity_	−0.281	0.123	0.022	-0.586
Model 3	BAI	0.366	0.132	0.005	0.915
	WCST_Rpe_	−0.008	0.159	0.962	-0.016
	BAI × WCST_Rpe_	0.099	0.141	0.479	0.301

BAI, the Beck Anxiety Inventory; SST_SSRT_, the stop signal reaction time; VWM_capacity_, visuospatial memory capacity; WCST_Rpe_, the rate of perseverative errors.

### 3.5. Moderate effects of executive function on the relationship between anxiety and specific checking symptom

Considering that executive function components were found has no moderate effect on the relationship between anxiety and compulsion symptoms. We further validate whether the VWM_capacity_ could moderate the relationship between anxiety and specific checking symptoms.

Hierarchical regression was utilized to explore the moderation effect of executive function. Data were first standardized. Gender, age, and education level were controlled as covariables. We used levels of anxiety (BAI) as independent variables, the severity of checking symptoms as dependent variables, and three indicators of executive function as moderators. In Model 1, SST_SSRT_ was used as the moderator. VWM_capacity_ was used as the moderator in Model 2, and WCST_Rpe_ was used as the moderator in Model 3.

The results of regression analysis showed that the main effect of anxiety on checking symptoms as measured by the OCI-R was significant (*p* < 0.010), but the main effects of SST_SSRT_ and WCST_Rpe_, and their interaction with anxiety level were not significant (*p* > 0.05). No moderating effects of SST_SSRT_ and WCST_Rpe_ were found between anxiety level and compulsive checking symptoms. However, the interaction between anxiety and VWM_capacity_ was significant in the VWM task (β = −0.281, *p* = 0.022) (Table 5).

**FIGURE 1:**
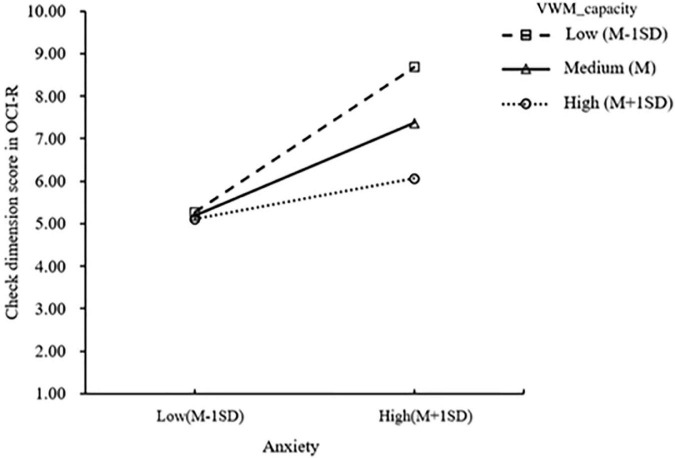
Simple slope test of visuospatial memory capacity (VWM_capacity_).

To further reveal the interaction effect, a simple slope test was performed. The results showed that the anxiety levels of patients with OCD with medium (M) and low (M-1SD) VWM capacity significantly predicted the severity of checking symptoms (*β_*L*_* = 0.171, *p*_*L*_ = 0.001; *β_*M*_* = 0.105, *p*_*M*_ = 0.009), and the effect was more significant in patients with low visuospatial memory capacity (Figure 1).

## 4. Discussion

The primary objective of the current study was to model the relationship between executive function, anxiety and checking behavior. The major results can be summarized as follows: (1) Compared with HC, patients with OCD showed more severe anxiety symptoms and significant impairment in VWM capacity, although there was no significant impairment in inhibition and set-shifting function; (2) Anxiety symptoms were positively (*r* = 0.361, *p* = 0.011) and VWM_capacity_ (*r* = −0.343, *p* = 0.016) was negatively related to compulsive checking behavior. (3) Anxiety symptoms showed significant direct predictive validity for compulsive checking behavior (*p* < 0.01), and the VWM function played a moderating role in the positive relationship between anxiety and compulsive checking behavior (β = −0.281, *p* = 0.022).

An initial objective of the study was to identify which components of executive function were significantly impaired in patients with OCD. According to the results of meta-analysis, effect sizes were medium in set-shifting, medium and medium-low in inhibition, while medium-to-large in visuospatial memory (43, 44). Our findings suggest that patients with OCD had impaired VWM capacity which is consistent with previous work in this area (5, 21, 45). Martínez-Esparza et al. (46) reported patients with OCD performed more poorly on measures of visuospatial working memory than control groups. Moreover, a meta-analysis showed that impairments in visuospatial memory are more pronounced in patients with OCD than are deficits in inhibition and set-shifting (43). The results that the observed no impairment of inhibition and set-shifting function in the current study is broadly consistent with previous research (20, 24). According to the cognitive theories of compulsive checking in OCD patients, the checkers are deficient in inhibiting misleading information and tolerating uncertainty, which may motivate reassurance-based checking of memory (7). Several studies have confirmed that (47, 48). Lambrecq et al. (5) used Corsi block-tapping test and delayed matching-to-sample task to find that it showed an opposite temporal direction in the relationship between abilities in visuospatial memory and uncertainty. However, there is no clear conclusion on whether pathological uncertainty leads to the decline of visuospatial working memory ability in patients with OCD, or whether compulsive checking behavior reduces confidence in memory and increases uncertainty, which still needs further research in the future.

Importantly, we also found significant correlations between VWM capacity and anxiety symptoms and compulsive checking behavior, which is consistent with previous reports that impaired VWM function and symptoms of anxiety are correlated (49, 50). Other researchers have also reported that anxiety consumes resources for goal-oriented behaviors (e.g., spatial attention, executive function), thus disrupting spatial working memory performance (51). According to the attention control theory, anxiety could occupy cognitive resources and interfere with the updating functions of the central executive system (52). The encoding of spatial information by VWM depends on the allocation of attention to the storage location, while anxiety is related to the consumption of central executive resources, which may undermine the efficient allocation of spatial attention (30).

A strong relationship between VWM and compulsive checking symptoms was reported in our study. Previous studies have showed that patients with OCD have lower reading and location working memory scores and longer checking times than HC, suggesting that insufficient VWM may increase uncertainty, leading to an increase in checking behavior (5, 48). This result may be explained by the cognitive theory of compulsive checking, which states that patients with OCD have deficits in inhibiting misleading information and tolerating uncertainty (7), and they have impaired memory of performing an action, and reduced confidence in their memory (48). The practice of repeated checking gives patients more information and reduces uncertainty. Thus, checking behavior can be viewed as a strategy to compensate for deficits in working memory (6, 7). Another possible explanation is that patients with OCD have deficits in balancing goal-directed and habitual behavior, while the ability to develop a plan individual to achieve a goal requires working memory function (10). Working memory could maintain goal-directed representations so individuals could respond to the problem without relying on previously learned associations (49). Moreover, functional neuroimaging evidence has shown that the orbitofrontal cortex (OFC), especially the medial OFC, appears to mediate executive control functions underlying the coordination of multiple working memory processes (53). This has been viewed as the neural basis of compulsivity behavior and is critical for the cognitive control of behavior (54, 55). Hence, it could conceivably be inferred that OFC dysfunction might disturb the capacity of VWM to hold goal-oriented representations, or reduce its capacity to maintain detailed characteristics of actions so that patients with OCD are required to resolve problems by relying on habitual checking behavior.

It is interesting to note that the moderate models showed that VWM had a valid moderating effect on the positive impact of anxiety on the severity of compulsive checking symptoms, and this effect has not been described previously. Patients with OCD with medium and lower VWM capacity showed a significant increase in checking symptoms with increased anxiety levels. However, this moderating effect was not observed in patients with higher visuospatial capacity. Thus, it seems that higher VWM ability might work as a protective factor for compulsive checking behavior in the face of substantial anxiety. A possible explanation for this might be that impaired VWM gives rise to an imbalance in the habit and goal-directed system and accordingly, leads to compulsive checking to alleviate anxiety.

Moreover, anxiety also interferes with the ability to filter out irrelevant information from VWM (56). Lower VWM capacity means that individuals are more disturbed (57), have less ability to regulate emotions (58), and also cannot appraise negative emotional stimuli well in an unemotional manner and require more neural resources in higher-order cognitive regions (59). There was also evidence showed that anxiety could alters self-control on memory, which change the self-confidence in memory, thus increasing the severity of compulsive checking symptoms (60). From a cognitive control framework (27), emotion regulation strategies (i.e., stopping, switching, and maintenance) are assumed to demand sound working memory updating ability. The impairment in stopping or switching ineffective emotion regulation strategies may lead to an overly rigid, inflexible, or repetitive use of regulatory strategies (61).

The results suggest that compulsive checking symptoms may be reduced by training to increase the capacity of VWM to improve working memory (6, 62). Shin et al. (63) performed a lateralized change detection task, and the results showed that the improvement during training was positively correlated with an increase in VWM capacity. However, studies of VWM training have not yielded consistent results, with some studies showing no significant increase in memory capacity (64), although this may be due to the use of different training methods. However, the results show that long-term training often shows a positive training effect although additional research is required to verify these findings.

## 5. Limitations

There are several limitations to this study. First, the sample size was relatively small, and whether our findings are generalizable to other patients with OCD requires validation. Second, the patients with OCD included in the study were not categorized into specific symptom dimensions and whether there are differences in the performance and moderation of cognitive function in different OCD subtypes needs further exploration. Third, this is an exploratory study. It may be due to the use of different measurement tools and paradigms, and the selection of different indicators, which may result in different results from other similar studies. It is suggested that future studies employ larger sample sizes and group OCD according to different subtypes to more thoroughly explore these relationships. Moreover, repeated validation should be performed using these measurement tools and paradigms. It can provide a foundation for the development of effective clinical interventions.

## 6. Conclusion

Taken together, the results presented in this paper offer an exciting opportunity for further research regarding how cognitive inflexibility and emotional factors interact to induce or maintain different subtypes or dimensions of OCD symptoms. To our knowledge, this is the first study to explore the moderating effect of executive function on the relationship between anxiety and compulsive checking behavior. That is, anxiety symptoms play a negligible role in explaining compulsive checking behavior in individuals with relatively strong VWM ability, but a substantial role in explaining compulsive checking behavior in individuals with relatively weak spatial visual working memory ability.

## Data availability statement

The original contributions presented in this study are included in the article/supplementary material, further inquiries can be directed to the corresponding author.

## Ethics statement

The studies involving human participants were reviewed and approved by Research Ethics Committee of Beijing Anding Hospital, Capital Medical University, Beijing, China. The patients/participants provided their written informed consent to participate in this study.

## Author contributions

PW: designed the study, performed literature searches, drafted the manuscript, critically reviewed, and revised the manuscript. ZY: performed literature searches, collected dated, and drafted the manuscript. TC: conceptualized the study, reviewed, and revised the manuscript. WC: collected data and revised the manuscript. XY, FM, and YL: contributed to collected data. ZL: contribute to conceptualized and designed the study, reviewed, and revised the manuscript, and as corresponding author. All authors contributed to the article and approved the submitted version.
